# Cell-Free DNA As Peripheral Biomarker of Alzheimer’s Disease

**DOI:** 10.14336/AD.2024.0329

**Published:** 2024-03-29

**Authors:** Sachi Khemka, Ujala Sehar, Pulak R Manna, Sudhir Kshirsagar, P. Hemachandra Reddy

**Affiliations:** ^1^Department of Internal Medicine, Texas Tech University Health Sciences Center, Lubbock, TX 79430, USA.; ^2^Public Health Department, School of Population and Public Health, Texas Tech University Health Sciences Center, Lubbock, TX 79430, USA.; ^3^Neurology, Departments of School of Medicine, Texas Tech University Health Sciences Center, Lubbock, TX 79430, USA.; ^4^Department of Speech, Language and Hearing Sciences, School Health Professions, Texas Tech University Health Sciences Center, Lubbock, TX 79430, USA.; ^5^Department of Pharmacology and Neuroscience, Texas Tech University Health Sciences Center, Lubbock, TX 79430, USA.; ^5^Nutritional Sciences Department, College of Human Sciences, Texas Tech University, 1301 Akron Ave, Lubbock, TX 79409, USA.

**Keywords:** Alzheimer’s disease, Alzheimer’s disease-related disorders, Cell-free DNA, DNA double-strand breakage, non-invasive early detection

## Abstract

Alzheimer’s disease (AD) and Alzheimer’s disease-related disorders (ADRD) are progressive neurodegenerative diseases without cure. Alzheimer’s disease occurs in 2 forms, early-onset familial AD and late-onset sporadic AD. Early-onset AD is a rare (~1%), autosomal dominant, caused by mutations in presenilin-1, presenilin-2, and amyloid precursor protein genes and the other is a late-onset, prevalent and is evolved due to age-associated complex interactions between environmental and genetic factors, in addition to apolipoprotein E4 polymorphism. Cellular senescence, promoting the impairment of physical and mental functions is constituted to be the main cause of aging, the primary risk factor for AD, which results in progressive loss of cognitive function, memory, and visual-spatial skills for an individual to live or act independently. Despite significant progress in the understanding of the biology and pathophysiology of AD, we continue to lack definitive early detectable biomarkers and/or drug targets that can be used to delay the development of AD and ADRD in elderly populations. However, recent developments in the studies of DNA double-strand breaks result in the release of fragmented DNA into the bloodstream and contribute to higher levels of cell-free DNA (cf-DNA). This fragmented cf-DNA can be released into the bloodstream from various cell types, including normal cells and cells undergoing apoptosis or necrosis and elevated levels of cf-DNA in the blood have the potential to serve as blood blood-based biomarker for early detection of AD and ADRD. The overall goal of our study is to discuss the latest developments in circulating cell-free DNA into the blood in the progression of AD and ADRD. Our article summarized the status of research on double-strand breaks and circulating cell-free DNA in both healthy and disease states and how these recent developments can be used to develop early detectable biomarkers for AD and ADRD. Our article also discussed the impact of lifestyle and epigenetic factors that are involved in DNA double-strand breaks and circulating cell-free DNA in AD and ADRD.

## Introduction

DNA damage and impaired DNA repair mechanisms are characteristic features of the aging process and are observed in various neurodegenerative conditions, including Alzheimer's disease (AD) [1, 2]. DNA damage can result in mutations and chromosomal aberrations, potentially causing cellular dysfunction or contributing to cancer formation. Interactions with specific DNA lesions have the potential to disrupt transcription and replication processes, leading to outcomes such as cell death, senescence, and the acceleration of the aging process [3]. The development of different neurodegenerative diseases entails errors within DNA repair mechanisms and the buildup of DNA damage. When DNA damage becomes extensive, the DNA damage response pathway can trigger cellular senescence and/or apoptosis. However, it remains to be established whether the increased levels of DNA damage in neurodegenerative disorders result from or contribute to the neurodegenerative events. Among potential DNA lesions, DNA double-strand breaks (DSBs) are infrequent but highly lethal. In neurons, DSBs are particularly harmful due to their limited DNA repair capacity compared to proliferating cells [4].

Similarly, the release of circulating cell-free DNA (cf-DNA) into the bloodstream is believed to be driven by apoptosis and/or necrosis, a process closely associated with cell damage and inflammation. Typically, macrophages are responsible for clearing up these fragments. However, in the context of cancer, excessive cell proliferation is thought to result in a higher accumulation of cf-DNA, as it surpasses the regular cleanup capacity of macrophages. Hematopoietic cells are the primary source of cf-DNA in healthy people. However, cancer patients’ tumor cells release their DNA, which is then contained in cf-DNA in varying amounts as "circulating tumor DNA" (ctDNA) [5]. Given that cf-DNA has been identified as originating from dying cells in cancer and entering the plasma, it is conceivable that cf-DNA may similarly leak into the bloodstream from degenerating neuronal cells [6].

Cell-free DNA has been identified in various bodily fluids, including blood, urine, and cerebrospinal fluid (CSF), and is believed to enter circulation through both active and passive processes. Cell death mechanisms, such as apoptosis and necroptosis, triggered by various internal and external factors, often result in incomplete digestion of intracellular nuclear and mitochondrial DNA, leading to the accumulation of circulating cf-DNA. Heightened concentrations of cf-DNA in the bloodstream of frail older individuals correlate with elevated cellular senescence and catabolic processes. Its detection in circulation is regarded as an indicator of cellular stress in diverse chronic conditions. [7, 8].

**Figure 1.**
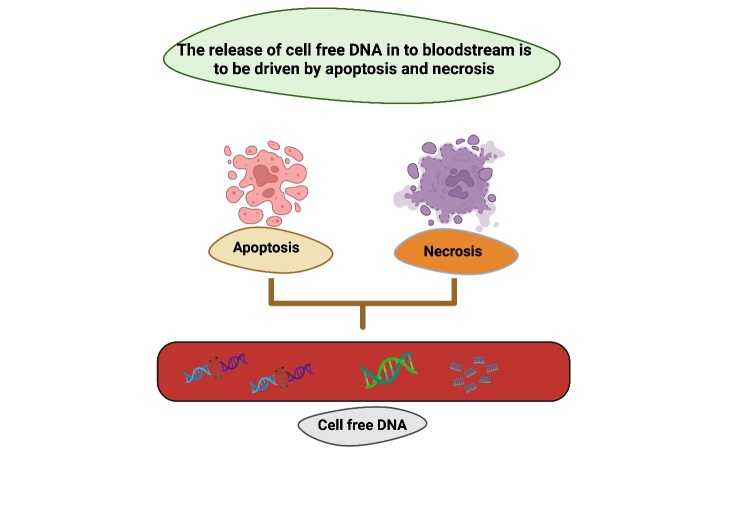
**DNA Damage with age**. cf-DNA is predominantly released into the bloodstream due to cell death because of apoptosis and necrosis, serving as a marker for cellular demise. Additionally, double strand breaks in DNA, arising from external factors like specific chemicals and radiation, as well as internal processes like DNA replication and repair, contribute to genetic alterations.

DNA damage accumulates with age due to exposure to environmental factors like UV radiation and oxidative stress. As the body's repair mechanisms become less efficient, genetic mutations and alterations in DNA structure increase, contributing to the aging process and elevating the risk of diseases such as cancer and neurodegenerative disorder, refer to Figure 1. Studying molecular mechanisms and enhancing DNA repair processes could provide insights into mitigating the effects of aging and promoting overall health. Several epidemiological investigations have revealed connections between Alzheimer's disease and related dementia and chronic inflammation. Previous research has established a strong correlation between dementia in later life and persistent inflammation that commences in middle age, indicating the potential for it to initiate or mediate neurodegenerative processes [9, 10].

A biological factor that could potentially contribute to chronic inflammation in Alzheimer's disease and related dementias is the presence of circulating cell-free DNA [8]. In theory, future non-invasive monitoring of AD could involve tracking neuronal, vascular, and inflammatory responses, as well as anatomical and functional changes in the brain [11]. This is supported by the understanding that the DNA released by cells in brain tissues contributes to the circulating pool of cf-DNA. While postmortem brain examination remains the definitive method for establishing AD pathology, present diagnostic protocols rely on clinical criteria and psychometric tests to determine the extent of cognitive impairment. Additionally, these guidelines may assess levels of Aβ and phosphorylated tau in cerebrospinal fluid (CSF) or utilize positron emission tomography (PET) brain imaging [12]. DSBs and cf-DNA can be explored as potential diagnostic tools for AD. Studies suggest that the presence and characteristics of DSBs and cf-DNA in various bodily fluids, including blood and cerebrospinal fluid, may provide valuable insights into the pathological processes associated with AD. Changes in DSBs and cf-DNA profiles, such as specific patterns or alterations, could serve as potential biomarkers for the early detection and monitoring of AD, offering a non-invasive and accessible diagnostic approach. Further research is underway to validate the utility of DSBs and cf-DNA as a diagnostic tool for AD.

The purpose of our article is to summarize recent developments in age-dependent DNA damage and circulating cell-free DNA in the blood in the progression of AD. We examined the impact of age and AD in double-strand breaks and circulating cell-free DNA and how these can be used as potential biomarkers for the early detection and monitoring of AD. We also discussed the lifestyle and epigenetic factors that are involved in double-strand breaks and circulating cell-free DNA in AD.

## Impact of Age and Lifestyle Factors on DNA Damage and Repair Capacity

Aging is an intricate and time-dependent biological process influenced by multiple factors, characterized by a gradual decline in both physiological and cognitive functions. Notably, typical behaviors, such as physiological brain activity when exploring a new environment, have been demonstrated to induce DNA double-strand breaks in neurons. [13]. This finding emphasizes the impact of lifestyle factors on DNA integrity and the importance of exploring potential therapeutic interventions to prevent the onset of neurodegenerative conditions associated with aging.

**Figure 2.**
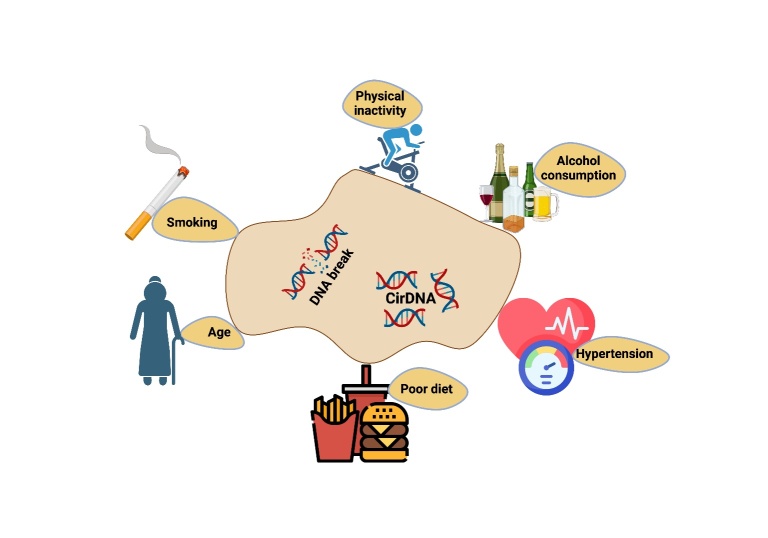
**Impact of lifestyle factors on DNA damage**. The expression levels of DNA damage biomarkers like double-stranded DNA breaks and circulating cell-free DNA are influenced by lifestyle factors. Elevated levels of these biomarkers are linked to lifestyle choices such as sedentary lifestyles, smoking/alcohol consumption, comorbidities, poor eating habits, and higher body mass indices.

The decline associated with aging leads to a diminished ability to respond to stressors, subsequently contributing to increased morbidity and mortality [14]. DNA damage related to age has been considered a potential factor in the process of aging, and these age-related alterations have been linked to various lifestyle factors [15]. Biomarkers of aging hold the potential for clinical applications, offering insights into disease outcomes and the responsiveness of older patients to invasive therapies. Moreover, there is a rising focus on developing molecular therapies and lifestyle interventions to decelerate the aging process. Nevertheless, assessing the effectiveness of these approaches poses challenges as it necessitates prolonged clinical studies to measure their impact on fitness and lifespan [16]. Lifestyle interventions prove highly effective in mitigating metabolic risk factors, genome instability, slowing disease progression, and alleviating disease-related side effects, refer to Figure 2. Moreover, these interventions contribute to decelerating the aging process. Genome instability, frequently linked to aging and the mentioned diseases is often triggered by inflammation and oxidative stress [17].

In an investigation carried out by Song and colleagues [16], they examined how lifestyle factors such as physical activity, body weight, and smoking affect the age-dependent manifestation of serum indicators for DNA damage, including Stathmin, CRAMP, n-acetyl-glucosaminidase, EF-1α, and chitinase, in comparison to established cellular aging markers like telomere shortening and p16INK4a upregulation in peripheral human blood. The study reveals that lifestyle choices and elements have a consistent impact on the expression of DNA damage indicators, regardless of age. Higher body mass indices and smoking were correlated with increased levels of DNA damage to biomarkers regardless of individuals' age. Conversely, exercise was linked to an age-independent decrease in the occurrence of DNA damage to blood biomarkers in humans.

The presence of DNA damage biomarkers exhibited a positive correlation with p16INK4a expression and a negative correlation with telomere length in T-lymphocytes from peripheral blood. These results offer experimental support for the influence of both aging and lifestyle factors on the accrual of DNA damage throughout the aging process in humans.

Yuwono and colleagues [18] reviewed 66 individual studies exploring cf-DNA levels concerning various factors such as exercise (both acute and chronic), occupational hazards, alcohol consumption, body mass index, smoking, hypertension, menstruation, circadian rhythm, stress, age, and biological sex. Despite methodological and technical inconsistencies among studies, the study identified acute exercise as a significant factor influencing cf-DNA levels. Considering the substantial increase in cf-DNA induced by acute exercise, the findings recommended routinely controlling physical activity before blood collection in study designs focusing on total cf-DNA levels. Another study aimed to evaluate DNA damage in lymphocytes using the cytokinesis-block micronucleus assay (CBMN) and assess cf-DNA in the blood of greenhouse workers exposed to pesticides, comparing them to matched controls residing in the same area. The CBMN assay was employed on peripheral blood lymphocyte samples obtained during various periods (spring and autumn) for each participant. Findings indicated a substantial increase in cf-DNA levels in the pesticide-exposed group compared to the control group, with no discernible seasonal fluctuation in free DNA quantity. Moreover, males demonstrated higher cf-DNA levels than females. The study concluded that pesticide exposure could influence DNA integrity through various mechanisms, and heightened cf-DNA levels may be considered a suggested biomarker for assessing pesticide exposure [19].

Various lifestyle factors and environmental exposures have been identified as potential sources of DNA damage. Alkylating agents, which are found in dietary components, tobacco smoke, and industrial processes, react with DNA bases, leading to the formation of mutagenic and carcinogenic lesions. Aromatic amines, which originate from sources such as cigarette smoke and industrial dyes, have the potential to transform into alkylating agents when activated. These agents can induce enduring DNA damage, causing alterations in DNA bases and shifts in genetic frames. Polycyclic aromatic hydrocarbons (PAHs) are a common carcinogen that is found in cigarettes. These PAH’s can generate reactive intermediates that react with DNA, leading to the formation of DNA adducts [20]. Other compounds like N-nitrosamines, 4-nitroquinoline 1-oxide, and estrogen also contribute to DNA damage through various mechanisms, including the formation of covalent adducts with DNA bases and induction of oxidative stress. Natural toxins, such as aflatoxins produced by microorganisms, can cause DNA damage by forming adducts with DNA bases, ultimately leading to depurination. Additionally, environmental stresses like extreme temperatures, hypoxia, and oxidative stress can induce mutagenesis and contribute to DNA damage, increasing the risk of various disorders, including cancer. Furthermore, items such as cosmetics, food preservatives, and plant protection agents, have been implicated in causing DNA damage. These commonly used items are connected to detrimental impacts on DNA integrity, underscoring the significance of comprehending and controlling exposure to them to alleviate health hazards related to DNA harm. Overall, a comprehensive understanding of the sources and mechanisms of DNA damage is essential for developing strategies to prevent and mitigate its adverse effects on human health [20].

Mitochondrial dysfunction leading to impaired bioenergetics and subsequent cell death has been observed in both dementia and frailty. A recent study proposes a common pathophysiology in aging contributing to physical and cognitive decline, with cf-mtDNA serving as potential mediators of these changes. The study found higher levels of short cf-mtDNA fragments were associated with lower global cognitive scores, while the ratio of short cf-mtDNA normalized to serum cf-nDNA was linked to increased mortality. Longitudinal analysis revealed that higher levels of long cf-mtDNA fragments were associated with greater frailty over time, while a higher integrity ratio (long cf-mtDNA relative to short cf-mtDNA) correlated with increased handgrip strength and improved global cognition, and decreased frailty scores in cross-sectional analysis. In conclusion, mitochondrial dysfunction, a primary theory of aging, is demonstrated to be associated with cognitive and physical trajectories over time, as indicated by the quantity and relative size of cf-mtDNA. This study underscores the utility of cf-mtDNA fragments in identifying and risk-stratifying older individuals prone to developing frailty and dementia [21].

Exercise training stands out as a therapeutic intervention that enhances health span by delaying the onset of age-related diseases and averting premature mortality. Telomere length, characterized by the 5′-TTAGGGn-3′ tandem repeats at the ends of mammalian chromosomes, serves as a key indicator of biological age [22]. Telomere length naturally decreases with age, and this progressive shortening can result in apoptosis, cellular senescence, or the oncogenic conversion of somatic cells, impacting an individual's health and lifespan. Shorter telomeres are linked to a higher rate of diseases and reduced survival rate. The occurrence of telomere shortening is influenced by lifestyle factors, and making better choices in diet and activities has the potential to either increase or decrease this occurrence rate. Optimal lifestyle choices can potentially slow down telomere shortening or, at the very least, prevent excessive attrition, thereby delaying the onset of age-related diseases and extending lifespan [23]. Consistent engagement in moderate-vigorous physical activity, adherence to dietary patterns abundant in vegetables and antioxidants, and the adoption of stress control techniques are associated with longer telomeres and enhanced oxidative response, marked by reduced levels of oxidative stress markers. Conversely, stress, obesity, smoking, and alcohol consumption exhibit a negative impact, leading to shorter telomeres and potentially contributing to premature aging [24]. Individuals who maintain a healthy lifestyle, incorporating elements such as balanced nutrition, caloric restriction, and regular physical exercise, tend to experience fewer age-associated epigenetic events. Healthy aging is linked to more compacted chromatin, reduced post-translational modifications (PTMs), and increased regulation by non-coding RNAs (ncRNAs) [25]. Magnesium supplementation has been associated with beneficial effects such as protecting against stress induced DNA damage. Research has shown that magnesium is involved in regulating cell cycle processes, apoptosis, and stabilizing nucleic acid structures. Magnesium also functions as a cofactor in the cell cycle. It is involved in processes such as DNA replication and repair, as well as the regulation of gene expression. Additionally, magnesium protects DNA from alkylation and other forms of damage [26]. Petrovic and colleagues conducted a study to investigate the impact of magnesium supplementation on DNA damage in young men with sedentary lifestyles and rugby players subjected to strenuous exercise. Results showed more DNA damage in rugby players as opposed to the sedentary students, both at baseline and when exposed to hydrogen peroxide. Magnesium supplementation significantly reduced DNA damage in both groups, particularly in rugby players. The article highlighted the value of magnesium supplementation for individuals with sedentary lifestyles as well as those engaged in vigorous physical activity [26]. Additionally, studies have found that a sedentary lifestyle has been linked to DNA damage. A study exploring the impact of an exercise regimen on DNA integrity revealed that engaging in physical activity may affect apoptosis levels, DNA damage, and telomere length. These findings hint at the potential of exercise in safeguarding genomic stability and mitigating oxidative DNA damage [27]. Another related research study conducted by Gill and colleagues focused on investigating the effect of a sedentary lifestyle on male fertility potential, particularly on sperm nuclear DNA integrity [28]. The study involved comparing semen samples from men who spent more than half of their working day in a seated position to those who spent less than 50% of their time in such positions. The study found that men with sedentary jobs exhibited a higher proportion of sperm with DNA fragmentation compared to those with less sedentary occupations. Further analysis revealed that sedentary workers were more than twice as likely to have high levels of sperm DNA damage compared to those in less sedentary occupations. The potential mechanism behind this detrimental effect could be linked to testicular heat stress, which may lead to failure in sperm chromatin remodeling during spermiogenesis [28].

## DNA Double-Strand Breakage

DNA double-stranded breaks (DSBs) occur when both strands of the DNA double helix are severed, often due to exposure to external factors such as ionizing radiation. This exposure can generate reactive oxygen species (ROS), leading to harm in biological compounds. Additionally, certain exogenously produced chemicals, like anti-cancer drugs, can induce strand breaks. DSBs can also arise when replication forks come across DNA lesions or repair intermediates. Repairing DSBs holds the possibility of genetic changes, allelic imbalance, and chromosomal restructuring, ultimately leading to either cell demise or the onset of cancer [29]. Interestingly, cells sometimes intentionally induce DSBs, particularly during meiosis, to facilitate gene shuffling in chromosomes [30].

Two primary pathways govern the repair of DSBs: homologous recombination and nonhomologous DNA end joining (NHEJ). The diverse origins of DSBs result in a variety of DNA and chemistries that necessitate repair. The significance of DSB repair cannot be overstated, as it is crucial for cell survival and genome stability. Eukaryotic cells have evolved intricate signaling networks to detect these DNA lesions and coordinate their repair processes [31].

Various techniques can be used to assess double-stranded DNA breaks, ranging from fluorescence microscopy and sequencing-based approaches to gel electrophoresis. Gel electrophoresis, particularly pulsed-field gel electrophoresis (PFGE), offers another avenue for detecting DNA breaks by identifying a fluorescent smear in treated samples. The comet assay, also known as single-cell gel electrophoresis, provides a means to estimate the quantity of DSBs [32]. Another method entails applying a DNA dye and utilizing a fluorescent microscope to observe nuclei and assess the displacement of DNA from the nucleus. This measurement is dependent on the number of DNA breaks per genome [32]. Alternatively, the quantitative DSB sequencing, qDSB-Seq, method gathers DSB frequencies per cell as well as their exact genomic coordinates through sequencing [33].

Double-strand breaks stand as severe forms of DNA damage implicated in various human diseases, including neurodegenerative disorders like AD [34]. The research underscores that DSBs can instigate chromosome breakage, genome instability, and neuronal loss in the context of neurodegenerative diseases [34]. The connection between DNA double-strand breaks and AD has been a focal point of investigation. Accumulation of DNA DSBs has been identified in the brains of individuals with AD, hinting at a potential association with the disease. Studies reveal that AD patients manifest signs of DNA repair deficits, with diminished levels of several proteins involved in DSB repair in their brains. This build-up of DSBs in neurons and astrocytes during the progression of AD is thought to contribute to neuronal degeneration and dysfunction [35] [36]. Alzheimer's disease involves an escalation in DSB formation, and it is coupled with a reduction in repair systems. In the context of various neurodegenerative diseases, there is a consistent manifestation of DNA Damage Response dysfunction. The cellular response to DSBs encompasses a broad array of signaling cascades, initiating with the recruitment of sensor proteins. Subsequently, through the involvement of ATM and other transducers, signals are transmitted extensively to numerous effectors, determining whether cell cycle arrest, DNA repair, or apoptosis should be enacted, contingent on the extent of the damage. DSBs are associated with neurodegeneration, primarily through the mechanism of apoptosis. In both Alzheimer's disease patients and R1.40 mice, the absence of nuclear CDK5 triggers neuronal cell cycle reentry, ultimately leading to apoptosis. The relocation of TDP-43 exacerbates DSB accumulation by inhibiting non-homologous end joining (NHEJ), a pathway responsible for repairing double-strand breaks in DNA [37].

It is estimated that between 10 to 50 DSBs occur in a single nucleated human cell during each cycle of cell division. This indicates the potential for the faulty integration of cell-free chromatin (cfCh) particles into healthy cells, originating from the numerous cells that die within the body daily [38]. The repetitive integration of cfCh into genomes may lead to significant outcomes such as DSBs, which are repaired inaccurately by NHEJ, ultimately resulting in apoptosis and the release of more cfCh. This results in a detrimental cycle of cfCh integration, DSBs, NHEJ, and increased cell death, potentially explaining the high rate of cell death in the body. Moreover, recent findings indicate that the incorporation of cfCh and subsequent DSBs trigger the activation of inflammatory cytokines. This suggests that the simultaneous occurrence of DSBs and inflammation over the course of a lifetime might be the root cause of aging, degenerative conditions, and cancer. [38]. A study by Nidadavolu and colleagues aimed to assess whether serum genomic cell-free DNA (cf-gDNA) is linked to physical and cognitive decline in older individuals. The findings of this study indicated that cf-gDNA fragments can effectively identify individuals at greater risk of developing dementia and experiencing worsening cognition and frailty [39].

The translocation of double-stranded damaged DNA into the bloodstream, contributing to the cell-free DNA (cf-DNA) population, encompasses a series of intricate mechanisms. Cellular damage, induced by factors like oxidative stress or radiation, may lead to double-strand breaks (DSBs). In instances of cellular damage, apoptosis, or programmed cell death, ensues, resulting in the packaging of DNA fragments into apoptotic bodies [40]. Subsequently, these apoptotic bodies, containing fragmented DNA, are released into the extracellular space. Macrophages, a type of immune cell, play a pivotal role in phagocytosing and clearing apoptotic bodies, breaking them down to release their contents [41]. The released DNA fragments, including damaged ones, enter the bloodstream as cf-DNA, with various cellular processes contributing to their release [42]. Size selection mechanisms may favor shorter cf-DNA fragments in the bloodstream [43]. Determining the precise origin of cf-DNA, whether from healthy or damaged cells, poses challenges, but specific genetic or epigenetic alterations in cf-DNA can offer insights into the cell types and the extent of damage. Many techniques enable the detection and analysis of circulating cf-DNA, holding potential for diagnostic, disease monitoring, and genetic alteration research purposes. In essence, damaged DNA, particularly from double-strand breaks, undergoes a complex journey into the bloodstream through cellular processes, ultimately contributing to the diverse population of cell-free DNA with clinical and research implications.

## Circulating Cell-Free DNA

After cell death, DNA is released into body fluids like blood plasma, forming cell-free DNA (cf-DNA), typically around 167 base pairs (bp) long. Cf-DNA, originating from various sources, holds unique diagnostic potential. In pregnancy, fetal cf-DNA in maternal plasma aids in diagnosing fetal genetic abnormalities via non-invasive prenatal testing. Cancer-derived cf-DNA detectable in blood plasma enables accurate cancer diagnosis using high-sensitivity methods like Next-Generation Sequencing (NGS) or droplet digital PCR (ddPCR). With a short half-life (< 2 hours), cf-DNA allows real-time disease burden monitoring, such as cancer relapse surveillance. Growing evidence supports the presence of brain-derived cf-DNA in cerebrospinal fluid (CSF) and blood plasma [44].

cf-DNA refers to the fragmented DNA released from cells into bodily fluids like blood, urine, and cerebrospinal fluid. It comes from both healthy and diseased cells and bears genetic information applicable for diagnostic and prognostic pursuits. In the realm of neurodegenerative diseases, extensive research has explored cf-DNA as a potential biomarker for early diagnosis and disease progression monitoring [45]. In pregnant women, cf-DNA of fetal origin is utilized for non-invasive prenatal diagnosis. Throughout pregnancy, a portion of the fetus's DNA circulates in the maternal bloodstream. cf-DNA screening is a procedure that analyzes this genetic material to evaluate the probability of specific chromosome disorders, such as Down syndrome (trisomy 21) [46]. While cf-DNA testing is a highly effective screening tool for detecting common chromosomal disorders, it does have limitations. It's crucial to note that a negative result does not completely rule out the possibility of the baby having a chromosomal disorder, or other conditions not covered by the test [47].

**Figure 3.**
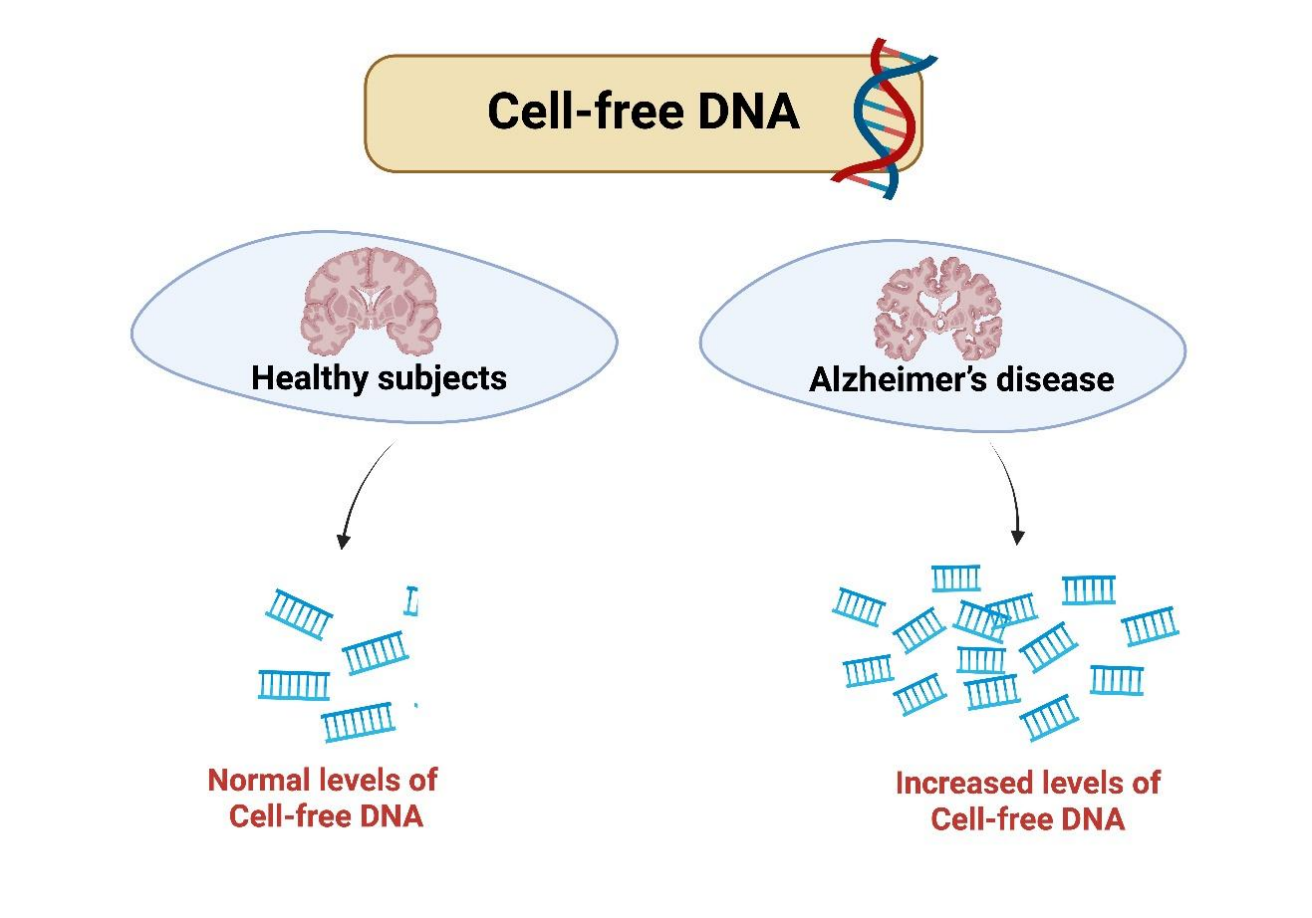
**Cell-free DNA in the blood bloodstream of healthy individuals versus individuals with dementia**. Circulating cell-free DNA, known for its role in immune stimulation and mortality prediction, is implicated in both dementia and frailty, reflecting mitochondrial dysfunction and cell death. The abundance and size of cell-free DNA fragments may signify the type of cell death, with long fragments indicative of necrosis and short fragments of apoptosis. The elevated concentrations of necrosis-associated cell-free DNA fragments and inflammatory markers in serum correlate with cognitive and physical decline and increased mortality risk.

Moreover, cf-DNA has shown promise in detecting and characterizing certain cancers and monitoring cancer therapy [46]. The utility of cf-DNA analysis extends notably to cancer diagnosis, selection of treatment options, and clinical follow-up. Its uses include predicting outcomes, identifying particular genomic changes, choosing targeted therapies, and continuous monitoring of treatment efficacy [48]. Particularly in cancer management, the analysis of circulating tumor DNA (ctDNA) as part of a liquid biopsy has gained attention. This method proves to be of use in screening, early diagnosis, therapeutic evaluation, and disease prognosis [49]. Explorations into cf-DNA-based approaches using methylation, mutations, or fragmentomes are underway for early detection of cancer. These endeavors hold the potential to enhance the performance and accessibility of current screening methods [50]. It is imperative to acknowledge that while cf-DNA testing shows promise for early cancer detection, its integration into routine clinical care necessitates further technological refinement and validation through clinical trials [51].

cf-DNA has also been linked to a heightened risk of dementia and alterations in physical and cognitive function, refer to Figure 3. The detection of cf-DNA leaking into plasma from dying cells in cancers [6] suggests a similar possibility in neurodegenerative conditions [52]. Neuronal apoptosis, triggered by factors such as oxidative stress or protein misfolding, could lead to the packaging of fragmented DNA into apoptotic bodies, like what is observed in cancer cells. Subsequently, these DNA fragments could be released into the blood circulation, contributing to the pool of cf-DNA. This phenomenon has potential implications for the study and diagnosis of neurodegenerative diseases, as analyzing cf-DNA in the bloodstream may provide insights into the genetic and epigenetic changes associated with neuronal degeneration. Further research in this area could enhance our understanding of the mechanisms underlying neurodegenerative processes and open avenues for non-invasive diagnostic approaches. Various studies indicate that cf-DNA fragments can be valuable in identifying individuals at a higher risk of developing dementia and experiencing cognitive decline [53]. A study conducted by Johns Hopkins Medicine discovered that DNA fragments may serve as early indicators of dementia and frailty, detectable through a simple blood test [54]. Further associations have been observed between inflammatory markers, circulating cell-free mitochondrial DNA, and physical and cognitive outcomes in older adults. These findings imply a potential connection between cf-DNA, inflammation, and cognitive decline [55].

In another recent study by Pollard and colleagues, they introduced a novel diagnostic strategy that employs comprehensive analysis of neuron-derived cf-DNA as a biomarker for the early detection of neurodegenerative diseases. Through the examination of Differential Methylation Regions (DMRs) between purified cortical neurons and blood plasma samples, the study identified robust biomarkers capable of accurately discerning between neuronal and non-neuronal cf-DNA. Targeted sequencing at the identified DMR locus revealed that employing a conservative cutoff of 5% of neuron-derived cf-DNA in blood plasma successfully identified 100% of patients diagnosed with AD, showcasing the promising potential for early disease detection. Notably, the proposed method effectively differentiated between patients with mild cognitive impairment (MCI) who later progressed to AD and those who did not, underscoring its prognostic capabilities [56].

## Measuring Cell-Free DNA

To measure cf-DNA in blood plasma and serum, various methods can be used, including manual and automated techniques. Several studies have compared different methods for isolating and quantifying cf-DNA from plasma and serum samples. For instance, a research investigation assessed the efficacy of six different commercial cf-DNA kits (comprising three magnetic bead-based, two spin column-based, and two automatic magnetic bead-based kits). The aim was to extract pure, high-quality cf-DNA from human plasma samples and analyze the quantity and size profiles of the cf-DNA extracts [57].

Another study aimed to compare the suitability of plasma and serum as specimens for circulating tumor DNA (ctDNA) analysis, utilizing high-sensitivity platforms like droplet digital polymerase chain reaction (ddPCR). Analyzing 119 matched plasma/serum samples from cancer patients, the study focused on comparing DNA profiles isolated from both specimens and examining the impact of differences on ctDNA measurements, aiming to identify the major contributors to any variations. The findings of the study revealed that serum exhibited an increased amount of large DNA fragments compared to plasma, while the distribution of cell-free DNA (cf-DNA) fragments (<800 bp) remained similar between the two. Importantly, ctDNA detection was less frequent in serum, and the fraction of KRAS-mutated ctDNA in serum was significantly lower than that in plasma [58].

The observed differences in ctDNA fractions correlated strongly with the abundance of large DNA fragments, as well as neutrophil and white blood cell counts. These results provide detailed insights into the distinctions between plasma and serum, using both DNA fragment sizing and ddPCR methodologies. The study advocated for the preference of plasma as the specimen type for ctDNA analysis as the dilution of tumor-derived DNA is minimized, optimizing the sensitivity of ctDNA analysis, and contributing to standardization in the pre-analytical process [58].

Additionally, a research study delved into the potential involvement of circulating cell-free DNA (cf-DNA) in regulating immune responses. The study focused on young, healthy volunteers, examining variations in telomeric sequence abundance in both plasma and serum. Additionally, the investigation explored the capacity of cf-DNA from these samples to co-activate TNF-α mRNA expression in monocytes. Utilizing qPCR, relative telomere length (T/S ratios) were determined in the serum, plasma, and whole blood of 36 volunteers. Paired plasma and serum samples were subjected to DNase treatment, allowing for the analysis of cf-DNA's contribution to TNF-α mRNA expression in the THP1 monocytic cell line. The findings unveiled significant disparities between serum samples and paired plasma in relative T/S ratios, with serum exhibiting higher values. Furthermore, both the total levels of cf-DNA and the estimated overall quantities of telomeres were significantly greater in serum when compared to plasma. Interestingly, the TNF-α mRNA expression in THP1 cells showed a significant increase after DNase treatment across all samples used for stimulation. Notably, the most elevated TNF-α mRNA expressions were observed following stimulation with DNase-treated serum samples. [59, 60].

**Figure 4.**
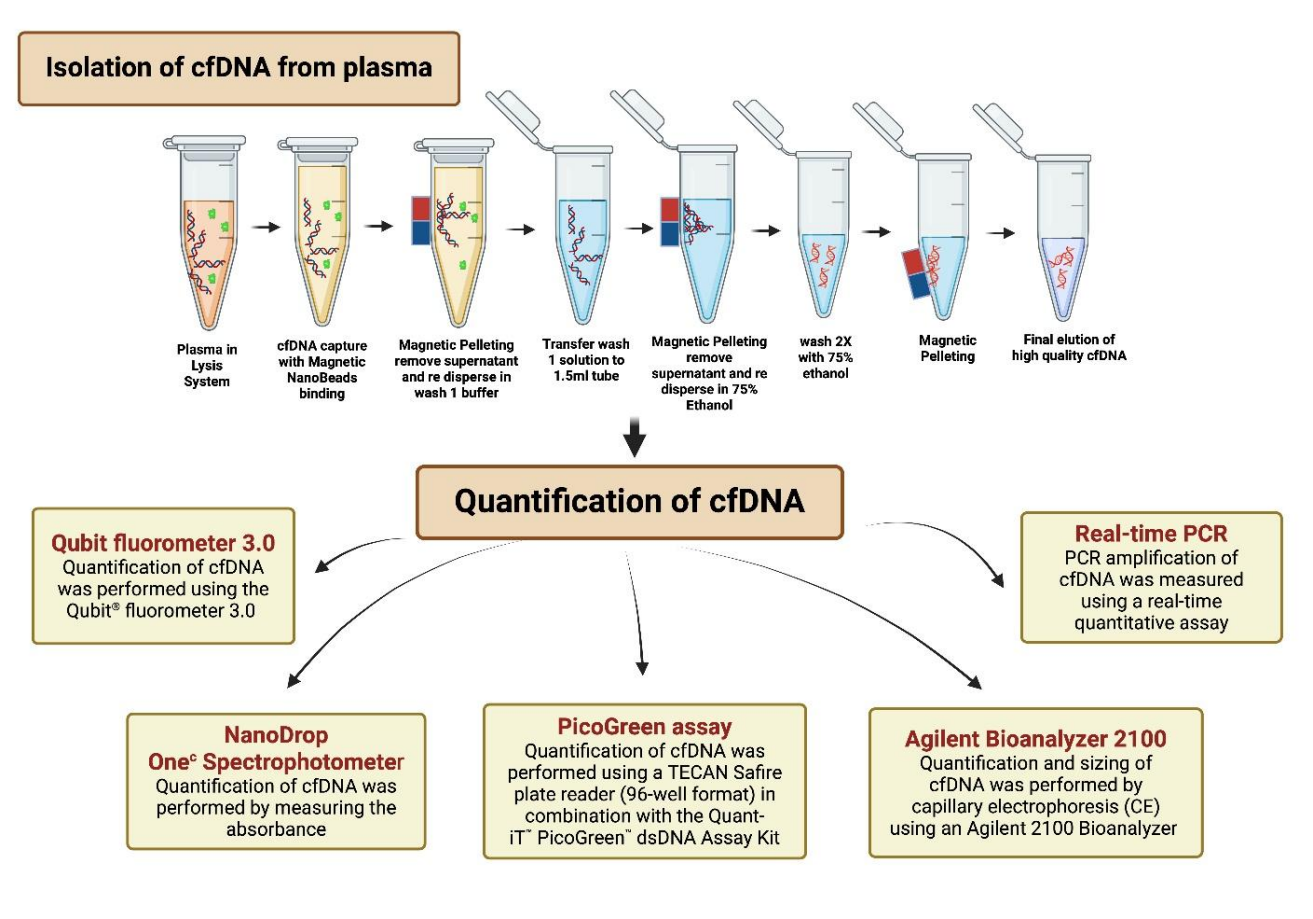
**To measure cell-free DNA in blood plasma and serum, here we showed a commercial cf-DNA kit (magnetic beads based) for extracting pure, high-quality cf-DNA from human plasma and serum**. Several methods have been described to quantify cf-DNA like Qubit fluorometer, NanoDrop, PicoGreen assay, Agilent bioanalyzer, and Real-time PCR.

The methods used in these studies include the isolation of cf-DNA from plasma or serum using commercial kits, DNA fragment sizing analysis, and digital droplet polymerase chain reaction (ddPCR) to assess the cf-DNA profiles and detect mutations. The studies also involved the use of qPCR to estimate the abundance of telomeric sequences. In summary, various methods, including commercial kits, refer to Figure 4, DNA fragment sizing analysis, ddPCR, and qPCR, are used to measure cf-DNA in blood plasma and serum. These methods provide valuable comprehension of the differences between serum and plasma in terms of cf-DNA content and the techniques used for isolation and quantification. Ensuring consistency and validation across methods for measuring cf-DNA is vital to its reliability as a biomarker. Standardizing protocols, conducting validation studies, and implementing quality control measures help minimize discrepancies. Cross-validation, proficiency testing, and continuous monitoring further enhance reliability and reproducibility, making cf-DNA a more robust biomarker for clinical applications.

## Ethnic Differences in Cell-Free DNA

Recent research has illuminated variations in cell-free DNA levels and utilization among different racial and ethnic groups. For instance, a study observed higher levels of cell-free total DNA in African American and Hispanic women during the first trimester, with an additional increase linked to higher BMI [61]. Another study revealed disparities in the adoption of cell-free fetal DNA (cffDNA) screening, particularly noting differences between Hispanic and White women [62]. Furthermore, investigations into the genomic landscape of cell-free DNA in men with advanced prostate cancer have unveiled distinctions based on race [63]. These findings collectively suggest noteworthy racial and ethnic variations in both cell-free DNA levels and the adoption of cf-DNA screening.

Cf-DNA has also proven to be a useful metric for pregnant women in terms of prenatal diagnoses and outcomes. Cell-free DNA (cf-DNA) obtained from the mother's blood can be utilized for non-invasive prenatal testing (NIPT), which can screen for chromosomal abnormalities such as trisomy 18, 13, and 21. This is possible because cf-DNA from the placenta, which is often reflective of fetal DNA, can be analyzed to assess the ratios of genetic material from different chromosomes. Furthermore, cf-DNA can also be used for non-invasive prenatal diagnosis (NIPD) to detect specific single-gene disorders in the fetus [64]. However, caution must be taken when using cf-DNA for prenatal testing as there are ethnic variations in these levels. A study investigated whether pregnant women with sickle-cell trait exhibited differences in cell-free DNA (cf-DNA) levels compared to healthy controls and whether there were ethnic disparities in cf-DNA levels between African and African-Caribbean populations compared to Northern European populations. The study revealed significant ethnic variations among the different ethnic groups, reinforcing the notion that ethnic factors should be considered in future cf-DNA research [65]. Continued research in this domain holds the potential to deepen our understanding of these differences and shed light on their implications for prenatal screening, cancer detection, and other clinical applications of cell-free DNA testing [46].

Research exploring ethnic differences in cf-DNA and epigenetic alterations reveals potential correlations with Alzheimer's disease. Ethnic groups may exhibit unique cf-DNA and epigenetic patterns influencing susceptibility to and progression of Alzheimer's. One study investigated the methylation of DNA and the variations among South Asian and European individuals in two UK-based cohorts. The study identified considerable disparities in blood DNA methylation across these different ethnicities, with revealing CpG sites linked to ethnicity dispersed across the genome. These differences are stable over time and are partially explained by variations in cell make-up. This study emphasized the impact of cell composition as a major driver of these distinctions [66]. Understanding these distinctions could enhance diagnostic precision and enable personalized interventions. Collaborative research across diverse populations is essential for understanding the complex interaction between environmental elements and genetic factors contributing to the onset of Alzheimer's disease.

**Figure 5.**
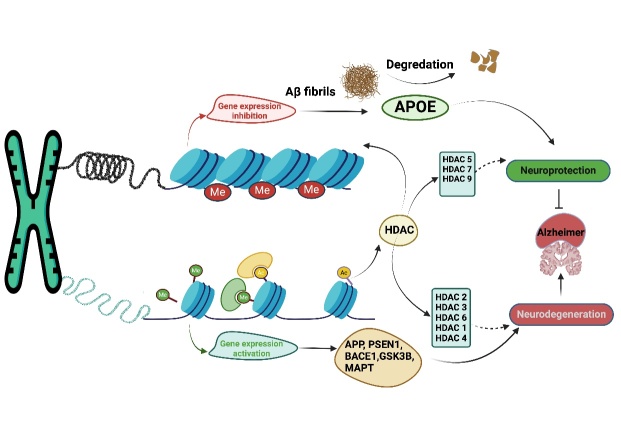
**Outline of the chromatin modifications changes underlying AD progress**. Chromatin remodeling including open (transcriptionally active) and closed (transcriptionally silenced) assemblies is composed of numerous architecture enzymes such as HATs, HDACs, and DNMT. In the pathogenesis of Alzheimer's disease, crucial genes subject to methylation modification include PSEN1, APP, APOE, and MAPT. The promoter APOE is silenced as well as hypermethylated in AD patients which results in the accumulation of Aβ fibrils in neurons and thus results in the progression of AD.

## Influence of epigenetic machinery on blood-based biomarkers of AD

Epigenetics is the study of heritable alterations in gene expression through both meiotic and mitotic processes, which do not affect the underlying core DNA sequence. Environmental stressors, both chemical and physical, along with diet, lifestyle choices, and pharmacological interventions, have the potential to influence the epigenome throughout an individual's lifespan, which modulates various pathophysiological processes [67]. Epigenetic dysregulation plays a crucial role in the progression of late-onset AD and other age-related complications and diseases, refer to Figure 5, implying the influence of epigenetic reprogramming in therapeutic interventions [68, 69]. The epigenetic landscape refers to the regulation of tissue-specific gene expression profiles in a variety of heritable changes, including histone modifications, chromatin compaction, and genomic imprinting. Aberrant epigenetic alterations progressively occur over time due to the age-associated progression of AD with the eventual reorganization of histone modifications, genomic imprinting, and chromatin modifying proteins to DNA damage, resulting in loss of neurons and ultimately neurodegeneration [69, 70]. Moreover, exposure to environmental toxins induces epigenetic changes linked to AD. The mechanism, involving DNA damage, remains unclear. Environmental exposures, causing DNA damage and reactive oxygen species, necessitate chromatin modifications for repair. Proteins involved in epigenetic silencing localize to damaged DNA sites. While chromatin responds transiently to DNA damage, chronic exposure may result in lasting epigenetic alterations. Understanding this mechanism is crucial for developing strategies to prevent or reverse these changes [71].

A key limitation for the development of therapeutic targets is to lack precise measures for early detection of cognitive impairment (MCI)/AD individuals from cognitively healthy subjects, because of time-consuming and expensive examinations, including magnetic resonance imaging and computerized tomography. As we know cf-DNAs encompass DNA, RNA, fetal DNA/RNA, long non-coding RNA (lncRNA), microRNA (miRNA), and mitochondrial DNA/RNA, and serve as potential biomarkers across various clinical conditions. The field is gaining prominence in precision medicine, offering valuable diagnostic, prognostic, and predictive insights. While DNA methylation in cf-DNA is widely utilized for personalized clinical diagnosis, the emerging roles of cfRNAs, such as miRNA and lncRNA, further contribute to the landscape of epigenetic biomarkers [72]. Under these circumstances, non-invasive peripheral biomarkers have frequently been utilized for the diagnosis of many disorders [73]. Accumulating evidence indicates that circulating microRNA (miRNA, ~20-25 nucleotides) levels in peripheral tissues are demonstrated to be closely associated with brain-region-specific alterations in AD patients [74-77]. A recent study has validated prior methylome findings in the promoter region of the PRLHR (prolactin-releasing hormone receptor) gene in the AD hippocampus [78]. Moreover, there is a correlation between methylation levels of PRLHR and β-amyloid deposits and P-Tau burden in hippocampal samples. The hippocampus in AD patients also displayed a significant decrease in PRLHR gene expression. Notably, these methylation differences were replicated in cf-DNA from the peripheral blood of an additional AD cohort, highlighting the potential utility of liquid biopsy techniques in identifying methylation variations in plasma cf-DNA of both AD patients and controls [78]. cf-DNAs exhibit promising potential as biomarkers for both the diagnosis and prognosis of a spectrum of diseases, including various cancers, obstetric conditions, autoimmune disorders, neurological ailments, mitochondrial diseases, and prenatal diagnoses [79].

Regulation of miRNA-based epigenetic mechanisms (currently over 2000 in mammals), involving post-transcriptional gene silencing along with histone modifications and DNA methylation, has been identified, and a number of them are implicated in the diagnosis and treatment of AD [80]. Besides, among several dozens of miRNAs studied in conjunction with AD, the majority of them are demonstrated to be altered in blood samples compared with controls, though the findings are often contradictory. Dysregulated expressions of miRNAs can be detected in the peripheral circulation such as blood, exosomes, and CSF of AD patients, which can serve as blood-based biomarkers [80, 81]. Employing a wide variety of approaches, Reddy and colleagues have demonstrated that miRNA-455-3p levels are elevated in the blood, CSF, and brain tissues of AD patients, and in AD mimetic conditions [74, 82-84]. Nonetheless, neuroprotective effects of miRNA-455-3p in APP processing and Aβ toxicity have been shown, in addition to the reversal of APP-induced mitochondrial and synaptic anomalies [83]. The efficacy and specificity of a large number of blood-based mRNA levels have been evaluated as AD biomarkers, and those with other epigenetic mechanisms, including DNA methylation in blood samples, could be useful for diagnosing MCI/AD [75, 80]. Overall, the functional comparison of brain tissues pertaining to AD and cognitively intact subjects and those of relevant blood samples indicated that methylation patterns are coordinately associated with blood specimens, hence, the latter could be an easily access reliable resource for reproducible biomarkers with clinical competence for early diagnosis of MCI/AD patients.

## Technological Advances in the Detection and Analysis of cf-DNA

### Sensitivity and Specificity

Several technological advances have been made to improve the detection and analysis of cf-DNA. This has significantly impacted diagnostic sensitivity and specificity and emphasizes the potential of cf-DNA as a valuable biomarker for diseases. Currently, low-plex methodologies, which involve analyzing a limited number of targets or parameters simultaneously, such as digital PCR, are being employed alongside massively multiplexed technologies like next-generation sequencing (NGS) for analysis of cf-DNA. NGS has advanced multiplexing capabilities and decreasing costs, and therefore, is widely used because of its high accuracy. Various techniques for enhancing specific genomic regions, including ligation, hybrid capture, and multiplex PCR, are used to improve the representation of genomic loci of interest. Nevertheless, persistent challenges related to PCR amplification errors, yield efficiency, and sequencing disparities impact the clinical sensitivity of cf-DNA assays. [85]. Detecting and quantifying cancer-specific mutations in cf-DNA from cancer patients poses significant challenges due to the low variant allele frequencies (VAFs) ranging from 0.01% to 10%. To address this, unique molecular identifiers (UMIs) have been widely adopted. UMIs serve to mitigate PCR and NGS errors, enabling the reliable detection of mutations at VAFs ≤0.1%. This is achieved by assigning a distinct DNA sequence to every individual DNA fragment within the cf-DNA sample, facilitating error correction during PCR amplification and sequencing. However, implementing UMIs requires higher sequencing depths and precise control over input cf-DNA amounts, leading to increased costs and complexity [85].

Nanopore sequencing, a method that allows for real-time DNA sequencing without the need for labeling or amplification, has shown the ability to detect cf-DNA and ctDNA with low concentrations present. This technique has also provided sensitive results [86, 87].

Furthermore, measures have been taken to increase the specificity of cf-DNA as a marker for disease. For studying cf-DNA for cancer diagnoses, normal cells have also been found to carry mutations associated with tumors, resulting in potential false-positive results in cf-DNA analysis. To avoid this, screening for multiple cancer-associated mutations simultaneously can enhance the detection of circulating tumor DNA (ctDNA). Integrating various biological information levels, such as DNA methylation and the presence of mutations, can further improve the precision of the analyses [88].

### Cost-Effectiveness

Novel nanopore sequencing techniques that do not require PCR amplification or bisulfite treatment have emerged for the comprehensive analysis of cf-DNA. These approaches enable the efficient characterization of cf-DNA even with minimal starting material, a limitation of using cf-DNA as a biomarker, resulting in a high yield of reads per sample while reducing costs compared to traditional methods [87, 89]. Additional progress in the exploration of cf-DNA for cancer detection and monitoring encompasses identifying fragmentation patterns, analyzing coverage of transcriptional start sites (TSS), sequencing T cell receptors, and examining cf-DNA for microbiome analysis. These approaches offer insights into tumor biology, immune response, and microbiome influence on cancer, potentially aiding in early detection, treatment selection, and treatment response assessment. These detection assays and analyses are cost-efficient techniques and hold promise in transforming cancer diagnostics and management by providing non-invasive and real-time monitoring options compared to traditional tissue biopsies [90].

## Limitations of the Utility of cf-DNA in clinical settings

Although cf-DNA shows significant potential as a blood-based biomarker for AD and offers promising applications in early detection of diseases as well as disease progression and treatment evaluation, additional investigation is required to thoroughly evaluate its efficacy and develop standardized protocols for its clinical application. One of the potential barriers to using cf-DNA as a biomarker is its low concentration. This makes it hard to measure and detect. Furthermore, given that cf-DNA is fragmented, it is more likely to be damaged by chemicals [85]. Additionally, no standardized approach has been determined to assess cf-DNA. cf-DNA is cleared from a patient’s blood at different rates that are contingent upon different biological and physiological factors, such as kidney function or plasma protein concentration, for example [88, 91]. Therefore, more research needs to be conducted to develop a standard protocol for measuring cf-DNA clearance. While the feasibility of utilizing cf-DNA analysis in clinical settings has been established, ensuring standardization and technical validation is crucial for its routine application. Additionally, challenges persist in clinical trial design, including small cohort sizes and the evolving complexity of genomic information, which affect the implementation and interpretation of cf-DNA analysis in clinical practice. Despite obstacles such as variations in sample collection methods and the selection of methodologies, there has been a noticeable increase in the number of clinical trials incorporating cf-DNA analysis. These trials signify ongoing efforts to overcome challenges and advance the utilization of cf-DNA as a valuable tool in cancer diagnosis and treatment [88].

## Conclusions and Future Directions

DNA damage emerges as a consistently implicated cellular process in the aging paradigm. Deficiencies in responding to DNA single- or double-strand breaks can lead to neurological diseases, underscoring the crucial role of DNA repair in maintaining neural homeostasis. The accumulation of DNA damage and compromised repair mechanisms plays a significant role in the pathogenesis of diverse neurodegenerative diseases. The DNA damage response pathway comes into play when damage is extensive, leading to the initiation of cellular senescence and/or apoptosis. However, the causative or consequential relationship between increased DNA damage levels and neurodegenerative events remains to be firmly established. DSBs represent the most severe form of DNA damage, demanding prompt repair by dedicated machinery. Mammalian cells employ various strategies, including homologous recombination, microhomology-mediated end-joining, and classic non-homologous end-joining, to recognize and repair chromosomal DSBs, depending on the cellular context and cell cycle phase. Beyond these repair pathways, the role of DNA damage response signaling, along with the involvement of heterogeneous nuclear ribonucleoprotein family proteins in repairing neuronal DSBs, has gained significance, particularly in age-associated neurological disorders. This paper introduced the hypothesis that DNA double-strand breaks lead to the release of fragmented DNA into the bloodstream, contributing to elevated levels of cf-DNA. This fragmented cf-DNA, originating from various cell types, including those undergoing apoptosis or necrosis, has the potential to serve as a blood-based biomarker for the early detection of AD and age-related neurodegenerative disorders (ADRD). cf-DNA offers distinct advantages over existing biomarkers for AD detection. Unlike traditional biomarkers such as amyloid-beta and tau proteins, cf-DNA provides a non-invasive method for AD diagnosis through blood plasma analysis. Additionally, cf-DNA's short half-life allows for real-time monitoring of disease progression, a feature lacking in many existing biomarkers. Moreover, cf-DNA's potential to detect brain-derived DNA fragments in CSF and blood plasma suggests promising advancements in early AD detection compared to current biomarkers. Studies on Cell-free DNA analysis show promise as a non-invasive blood-based biomarker for early Alzheimer's detection, with unique patterns indicating pre-symptomatic pathology. Its accessibility and potential for large-scale screening align with the liquid biopsy paradigm, offering a cost-effective approach for timely interventions. However, further research is needed to validate and establish its reliability for widespread clinical use.
